# Sex composition of living children in a matrilineal inheritance system and its association with pregnancy intendedness and postpartum family planning intentions in rural Ghana

**DOI:** 10.1186/s12978-018-0616-2

**Published:** 2018-11-09

**Authors:** Sebastian Eliason, Frank Baiden, Derek Anamaale Tuoyire, Kofi Awusabo-Asare

**Affiliations:** 1 Department of Community Medicine, University of Cape Coast, Cape Coast, Ghana; 2 Centre for Health Research and Implementation Support, Accra, Ghana; 3 Department of Community Medicine, School of Medical Sciences, University of Cape Coast, Cape Coast, Ghana; 4 Department of Population and Health, University of Cape Coast, Cape Coast, Ghana

**Keywords:** Sex composition, Unintended pregnancy, Postpartum family planning intention

## Abstract

**Background:**

Sex composition of living children within the context of “sex preference” and its association with various reproductive health outcomes has been extensively studied in South and South East Asia. Although sex preference has been observed in sub-Saharan Africa, there is paucity of research on sex composition of living children and its association with reproductive health behaviours and outcomes, particularly in a matrilineal inheritance system. The purpose of the study was to explore the existence of sex preference in a matrilineal inheritance system. Specifically, the study sought to better understand the issues by examining the sex composition of living children and how it is associated with reproductive outcomes such as pregnancy intendedness and intention to use postpartum family planning among women in a matrilineal area of Ghana.

**Methods:**

This was a cross sectional study conducted at four selected health facilities in the Mfantseman municipality of the Central Region of Ghana. Out of the 1914 pregnant women attending antenatal clinic selected using total enumeration, from 2nd January to 30th April 2012, 1091 with living children and complete socio-demographic data were recruited for this study. Descriptive, chi square and multivariate logistic regression analyses were conducted.

**Results:**

The mean age of the 1091 respondents in this study was 28.2 ± 6.0 years with mean gestational age of 26.7 ± 6.6 weeks. Whilst 78% of the women had at least a son, 71% had at least a daughter, with those having only sons exceeding those with only daughters by 6.3%. Also, majority of the women with more sons than daughters did not intend their current pregnancies. These observations, coupled with a sex ratio of 109 males to 100 females, inferred the existence of son preference. The levels of unintended pregnancy and intention to use postpartum family planning were high (70% and 78% respectively). There was an association between sex composition of living children and unintended pregnancy but no association between sex composition of living children and intention to use postpartum family planning. Women with only sons were 50% less likely to have unintended pregnancies compared to those with equal number of sons and daughters [AOR 0.5, 95% CI (0.3–0.8)]. Similarly, women over 30 years were 80% less likely to have unintended pregnancies compared to those 15–19 years [AOR 0.2, 95% CI (0.1–0.6)]. The women with parity of 5 or more and resident in Anomabo were more likely to have unintended pregnancy compared to those with parity of up to two [AOR 3.8, 95% CI (1.7–8.59)] and those resident in Saltpond [AOR 1.8, 95%CI (1.1–2.8), respectively. In addition, the women resident in Anomabo were more likely to have intention to use postpartum family planning compared to those in Saltpond [AOR 1.8, 95% CI (1.0–3.3)].

**Conclusion:**

There was persistence of more sons than daughters born in a predominantly matrilineal inheritance system and sex composition of living children had significant association with pregnancy intendedness but not with intention to use postpartum family planning.

## Plain English summary

Sex composition of living children within the context of “sex preference” and its association with various reproductive health outcomes has been extensively studied in South and South East Asia. Although sex preference has been observed in sub-Saharan Africa, there is paucity of research on sex composition of living children and its association with reproductive health behaviours and outcomes, particularly in a matrilineal inheritance system. The purpose of the study was to explore the existence of sex preference in a matrilineal inheritance system. Specifically, the study sought to better understand the issues by examining the sex composition of living children and how it is associated with reproductive outcomes such as pregnancy intendedness and intention to use postpartum family planning.

This was a cross sectional study conducted at four selected health facilities in the Mfantseman Municipality of the Central Region of Ghana. Out of the 1914 pregnant women attending antenatal clinic selected using total enumeration, from 2nd January to 30th April 2012, 1091 with living children and complete socio-demographic data were recruited for this study. Descriptive, chi square and multivariate logistic regression analyses were conducted.

The mean age of the 1091 respondents in this study was 28.2 ± 6.0 years with mean gestational age of 26.7 ± 6.6 weeks. Whilst 78% of the women had at least a son, 71% had at least a daughter, with those having only sons exceeding those with only daughters by 6.3%. Also, majority of the women with more sons than daughters did not intend their current pregnancies. These observations, coupled with a sex ratio of 109 males to 100 females, inferred the existence of son preference. The levels of unintended pregnancy and intention to use postpartum family planning were high (70% and 78% respectively). There was an association between sex composition of living children and unintended pregnancy but no association between sex composition of living children and intention to use postpartum family planning. Women with only sons were 50% less likely of having unintended pregnancies compared to those with equal number of sons and daughters [AOR 0.5, 95% CI (0.3–0.8)]. Similarly, women over 30 years were 80% less likely to have unintended pregnancies compared to those 15–19 years [AOR 0.2, 95% CI (0.1–0.66)]. The women with parity of 5 or more and resident in Anomabo were more likely to have unintended pregnancy compared to those with parity of up to two [AOR 3.8, 95% CI (1.7–8.6)] and those resident in Saltpond [AOR 1.8, 95%CI (1.1–2.8) respectively. In addition, women resident in Anomabo were more likely to have intention to use postpartum family planning compared to those in Saltpond [AOR 1.8, 95% CI (1.0–3.3)].

In conclusion, there was persistence of more sons than daughters born in a predominantly matrilineal inheritance system and sex composition of living children had significant association with pregnancy intendedness but not with intention to use postpartum family planning.

## Background

Sex composition of living children and its association with various reproductive health outcomes within the context of preference for one sex or the other, has been extensively studied in South and South East Asia, where an estimated 30–70 million women are believed to be unaccounted for [1], and sex ratios at birth as high as 130 males to 100 females have been observed [2]. Among the reasons identified to account for the situation are: the patriarchal nature of the society; the attitude that sons are more important and valuable than daughters in carrying out important religious roles; that sons have the right to inherit land; that sons support aged parents and perpetuate the family name [3].

According to a joint statement issued by five United Nations (UN) organizations including the Human Rights Office (OHCHR) in 2011, there is huge pressure on women to produce sons, which not only directly affects women’s sexual and reproductive lives with implications for their health and survival, but also puts women in a position where they must perpetuate the lower status of girls through son preference [2].

A study by Chaudhuri found that in India, women with more sons than daughters were less likely to progress to higher parities than were women with more daughters than sons [4]. Other studies in South East Asia have shown that women with more sons are more likely not to want any more children and more likely to use modern family planning methods [5, 6].

In sub-Saharan Africa, balance has been observed as the most common type of preference. Of 28 sub-Saharan African countries reviewed in a study by Fuse, balance was found to be the most popular preference in 24 countries [7]. The study further revealed that though son preference is observed in every sub-region within sub-Saharan Africa, it appeared to be particularly prevalent in West Africa, especially Mali, Senegal and Burkina Faso. Daughter preferences have also been observed in some West African countries including Ghana, Liberia and Sierra Leone [7]. In Nigeria, a study showed that parental gender preferences did influence fertility behaviour and was largely shaped by social institutions [8].

In the studies cited above, one of the identified factors influencing son preference in sub-Saharan Africa is the patriarchal nature of society. However, there are very few studies on sex preference in matrilineal societies. One of the questions then is whether there will be preference for one sex or the other in matrilineal areas.

In the southern sector of Ghana where most of the Akans live [9], three main types of kinship systems exist: matrilineal, patrilineal and dual [10–12]. The matrilineal Akans trace their lineage through the female. Although matrilineal, it has elements of patriarchal structures. For instance, in most cases, the heads of matriclans are males. The other two major groups in the southern sector of the country, the Ewe and Ga-Adangbe, are patrilineal and patriarchal [12].

Under the matrilineal system, the relations consist of uterine siblings (i.e those born of the same mother but not necessarily the same fathers) [12]. It will be expected that in such a matrilineal system, there will be preference for daughters who will contribute to the matriclan or for balance, to ensure continuity. As noted by Fuse, the pattern for Ghana was for balance (47%), daughter preference (21%) and son preference (19%) [7]; this was based on national data and did not consider these differences in lineage systems. A commonly used approach to studying sex preference investigates the association between family sex composition and actual fertility behaviours, such as family planning use or fertility desire [3]. The purpose of this study was to explore the existence of sex preference in a matrilineal inheritance system. Specifically, the study sought to better understand the issues by examining the sex composition of living children and how it is associated with reproductive or fertility outcomes such as pregnancy intendedness and postpartum family planning intentions, among predominantly matrilineal women in a rural setting in Ghana.

## Methods

It was a cross-sectional study conducted between 2nd January and 30th April 2012 at four health facilities in the Mfantseman Municipality (predominantly rural in nature) of the Central Region of Ghana, namely the Saltpond Municipal Hospital, Mankessim Health Centre (both primary level and semi-urban) and the Biriwa and Anomabo Health Centers (both primary level and rural). These health facilities were selected out of seven public health facilities because they were the main facilities in the municipality that carried out almost all the reproductive, child health and nutrition activities, had relatively high numbers of antenatal visits and deliveries [13], and also gave a good mix of semi-urban and rural settings. Using total enumerative sampling technique, all pregnant Ghanaian women, aged 15–49 years, living in the municipality and attending antenatal clinic at any of the selected health facilities at designated antenatal times, were selected for interview within the premises of the health facilities using a five-page questionnaire. Questionnaire administration was carried out by trained field assistants and supervisors mainly from the Mfantseman Municipal Health Directorate.

The questionnaire was constructed using information mainly from questionnaires of the Ghana Demographic and Health Survey 2008. Review of the questionnaire was carried out with support of the Mfantseman Municipal Director of Health Services, Medical Superintendent of the Saltpond Municipal Hospital, the Director of Family Health Division, Ghana Health Service and Academic Senior members of the University of Cape Coast. Questions related to both explanatory and outcome variables were asked. Those related to explanatory variables included socio-demographic characteristics of respondents, the number of living children and sex of the children, issues pertaining to the nature of relationship between respondents and their male partners and awareness and ever use of various family planning methods. Those related to the outcome variables included respondents’ reproductive history, including the assessment of the current pregnancy as intended or unintended, and the intention to adopt postpartum family planning (PPFP).

Based on an estimated target population (N_f_) of 4218 (an average number of women attending antenatal clinics per quarter from 2007 to 2010 in the municipality) [13], and the assumption that 50% of pregnant women intended to adopt postpartum family planning, within a margin of error of 3%, a minimum sample size S_f_, was estimated as follows: For a finite population, the sample size S_f_, was estimated by the formula S_f_ = A / [1 + (A-1) / N_f_] [14], where A is given by [Z*P*(1-P)] / C^2^; N_f_ = estimated target population; Z = Z value (1.96 for 95% confidence); P = proportion of pregnant women who intended to adopt postpartum family planning; and C = margin of error [15]. A = [1.96^2^(0.5)(0.5)]/0.03^2^ = 1067; S_f_ = 1067/[1 + (1067–1)/4218] = 852; (approximated to 900). For effective antenatal care and early identification and management of complications, it was important and advisable for all women to report to antenatal clinic as soon as they noticed they were pregnant [13]; implying that all pregnant women were assumed to have registered in the first trimester of pregnancy. Evidence from the Ghana Health Service, Central Regional Family Health Report, 2011, showed that this was not the case: only 43% registered in their first trimester, whilst the remainder registered in the second or third trimester [13]. It was also recommended that all antenatal registrants delivered at the health facilities at term to ensure adequate supervision during delivery by skilled personnel. However, the same report showed that only 44% of them delivered at the health institutions within the municipal area [13]. This implied a default rate of close to 60%. To take care of defaults and late registrations, the minimum sample size (S_f_ = 900) computed was doubled to 1800 with an additional 10% mark-up for women who declined to be interviewed. The estimated total sample size S_T_ was 1980.

**Fig. 1:**
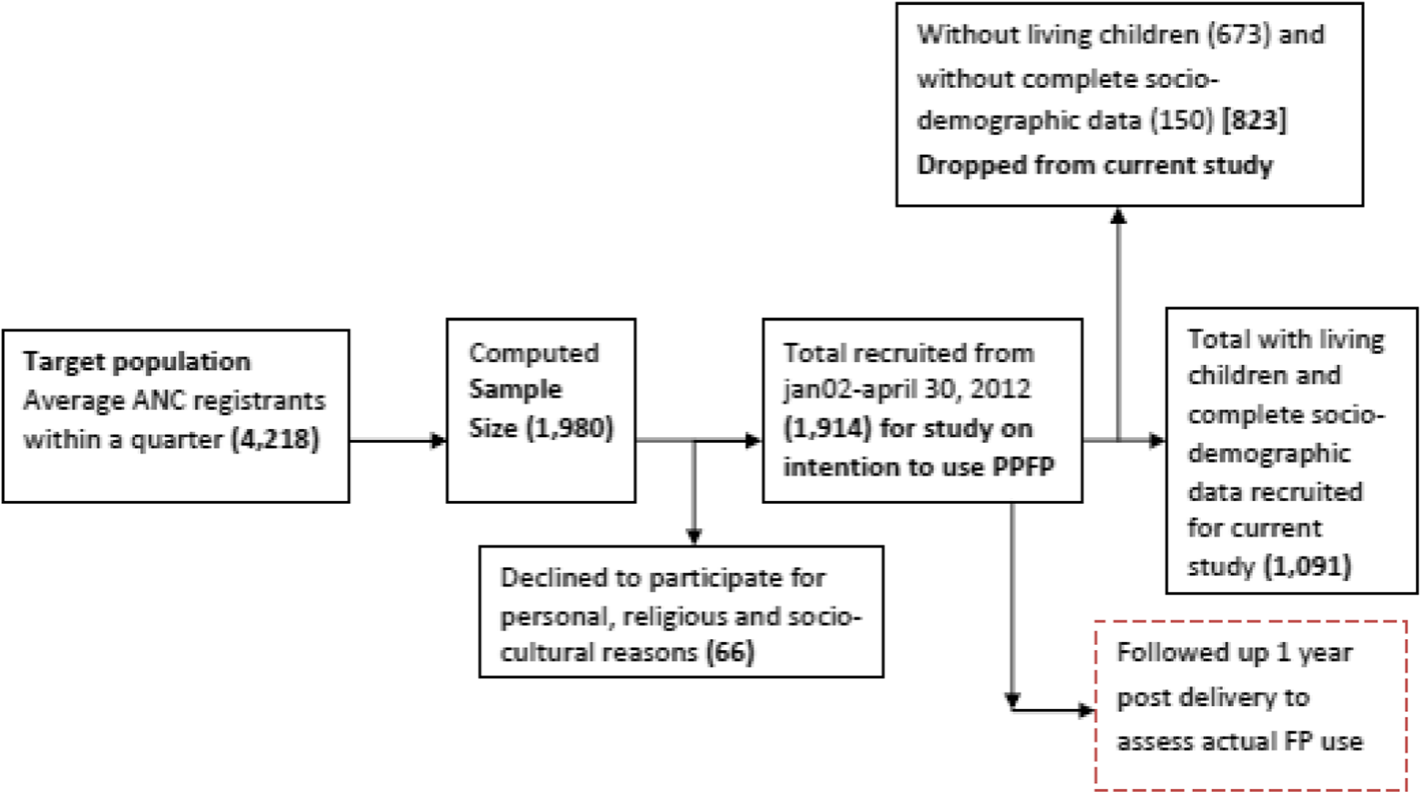
Flow diagram showing how participants were recruited for current study Source: Field work 2014

It was estimated from municipal records [13] that the ratio of average antenatal visits in the municipal hospital to those of a health centre was 3:1. Therefore, the estimated sample size for each health centre is given by S_h_ = S_m_/3; but the computed sample size for the Saltpond municipal hospital was approximately 990; implying that of each health centre is approximately 330. The total estimated sample size (S_T_) is given by S_T_ = Sm + 3Sh = 1980. Within the period of the study, 1914 pregnant women were interviewed with distribution as follows: Saltpond municipal hospital = 968(response rate, RR-97.8%); Mankessim health centre =289(RR-87.6%); Anomabo health centre =327 (RR-99.1%); Biriwa health centre = 330(RR-100%). Disparities in response rates occurred because some qualified clients declined interview for socio-cultural, religious and personal reasons. For the purposes of this study however, women with living children at the time of the survey and whose socio-demographic data were complete (1091) were recruited from the total sample of 1914 and characterized using total number and sex of children. Figure 1 shows a flow diagram of the selection process. At the end of each interview, field assistants and/or supervisors checked the questionnaires for completeness and consistency before the clients were allowed to leave.

Each of the selected health facilities was assigned a peculiar code to allow for easy identification and tracking if errors and inconsistencies were detected. For data entry, a coding manual was developed in order to ensure consistency. The data were double-entered using the EPI-DATA and verified. The cleaned data were exported into STATA/IC (version 11.2) for analysis.

The main outcomes of interest in this study were “unintended pregnancy” and “intention to adopt family planning postpartum”. Each of the outcome variables was constructed as a binary outcome and defined as follows: unintended pregnancy (denoted as ‘1’) - any pregnancy that was unwanted (i.e. that occurred when no children, or no more children were desired) or mistimed (i.e. that occurred earlier than desired). An intended pregnancy (denoted as ‘0’) was defined as any pregnancy that was wanted at the time it occurred; intention to adopt contraceptives postpartum is defined as intending to adopt a family planning method in order to avoid getting pregnant too soon after delivery; ‘1’ denoting intention to adopt and ‘0’ denoting intention not to adopt postpartum family planning.

The key explanatory variable of interest in this study was sex composition of children. This was based on the sex and number of living children. Sex composition of children was categorized into five groups as follows: (a) only daughters; (b) only sons; (c) more daughters than sons; (d) more sons than daughters; and (e) equal numbers of daughters and sons. In addition, some key demographic characteristics considered in the analysis included age, marital status, education, parity, gravidity, religion, occupation and area of residence.

Descriptive analyses of the explanatory variables were conducted using simple proportions, means and standard deviations. Chi square statistics was used to explore the association between explanatory variables (sex composition and key socio-demographic characteristics) and the outcome variables (unintended pregnancy and intended PPFP use). A significant association was said to exist if *P* < 0.05. This significance level was selected based on its application in previous studies and because it provides a fair chance of picking up those effects which are large enough to be of scientific interest.

Controlling for key socio-demographic characteristics, a multivariate logistic regression analyses was conducted to determine if sex composition was independently associated with pregnancy intendedness and intention to use postpartum family planning. Strength of association was determined by computing odds ratios and confidence intervals. Significance levels were determined at *P* < 0.05.

Ethical approval was obtained from the Ethics Review Committee of the Ghana Health Service (GHS) (GHS-ERC: 14/09/11). Institutional approval was also obtained from the Municipal Health Directorate (MHD) and the heads of the facilities where the surveys were conducted. Written informed consent was obtained from each participant before the administration of questionnaires.

## Results

### Descriptive analysis

The mean age of the 1091 respondents interviewed in this study was 28.2 ± 6.0 years, with majority of them (30.1%) aged 25–29 years (Table 1). The mean gestational age was 26.7 ± 6.6 weeks. The average number of children per woman was 2.0 ± 1.0 and sex ratio of 109 males to100 females (consisting of 861 males and 792 females). The women who had only sons constituted 28.7% whilst 22.4% had only daughters. Of those who had sons and daughters, 13.8% had more daughters than sons, 15% had more sons than daughters and 20.1% had equal number of sons and daughters (Table 1). This implies that respondents who had at least a son and a daughter constituted 78% and 71% respectively. Those who had only sons exceeded those with only daughters by 6.3%.

**Table 1:** Characteristics of participants and their association with unintended pregnancy and intention to use postpartum family planning (PPFP)

	Sample		Unintended Pregnancy	Intention to use PPFP
Characteristic	N	%	%	%
Sex Composition				
Only daughters	244	22.4	65.2	77.9
Only sons	313	28.7	58.8	75.4
More daughters than sons	151	13.8	78.1	83.4
More sons than daughters	164	15.0	84.1	78.0
Equal number of sons and daughters	219	20.1	72.6	77.2
X^2^(*P* value)	–	–	42.0(0.000)	3.9(0.421)
Age				
15–19	36	3.3	80.6	72.2
20–24	293	26.9	68.9	78.8
25–29	328	30.1	70.1	78.7
30–34	237	21.7	63.7	77.2
35–39	146	13.4	71.9	75.3
45+	51	4.7	80.4	80.4
X^2^(P value)	–	–	9.2(0.102)	1.7(0.885)
Educational level				
No education	289	26.5	75.4	78.2
Primary	268	24.6	71.3	82.5
Middle/JSS	447	41.0	68.0	78.3
SSS/SHS/Vocational	65	6.0	53.8	61.5
Tertiary	22	2.0	45.5	54.5
X^2^(P value)	–	–	19.2(0.001)	20.3(0.000)
Religion				
Christian	1009	92.5	69.8	78.2
Muslim	53	4.9	60.4	67.9
Others	29	2.7	75.9	82.8
X^2^(P value)	–	–	2.7(0.263)	3.5(0.174)
Parity				
1—2	684	62.7	63.9	76.3
3—4	307	28.1	75.6	79.8
5+	100	9.2	89.0	82.0
X^2^(P value)	–	–	33.4(0.000)	2.6(0.271)
Residence				
Saltpond	259	23.7	64.5	80.3
Biriwa	132	12.1	75.8	78.0
Anomabo	187	17.1	77.5	88.8
Mankessim	305	28.0	65.9	69.5
Others	208	19.1	69.7	76.9
X^2^(P value)	–	–	13.1(0.011)	26.2(0.000)

### Characteristics of women and their association with unintended pregnancy and intention to use postpartum family planning

Unintended pregnancies were generally high among the women (70%). An association existed between unintended pregnancy and sex composition of children (X^2^ = 42.0, *P* < 0.001); educational status (X^2^ = 19.2, *P* < 0.01); parity (X^2^ = 33.4, *P* < 0.001); place of residence (X^2^ = 13.1, *P* < 0.05), occupation (X^2^ = 32.3, P < 0.001) and marital status (X^2^ = 21.6, *P* < 0.001) [Table 1]. The highest levels of unintended pregnancy were among women with: more sons than daughters (84.1%); no education (75.4%); parity five or more (89%); residence at Anomabo; farming as occupation (80%); and single women (81.8%) [Table1].

Similarly, a high percentage of women (78%) expressed intention to use postpartum family planning. An association existed between women with intention to use postpartum family planning and educational status (X^2^ = 20.3, *P* < 0.001), place of residence (X^2^ = 26.2, P < 0.001); occupation (X^2^ = 17.1, *P* < 0.01) and marital status (X^2^ = 21.0, *P* < 0.001). There was however, no association between intention to use postpartum family planning and sex composition of children. The women with primary education, resident in Anomabo, farmers and those cohabiting, expressed the highest levels of intention to use postpartum family planning (Table 1).

### Multivariate logistic regression analyses of sex composition on unintended pregnancy and intention to use postpartum family planning

Controlling for socio-demographic characteristics, women with only sons were less likely to classify their pregnancy as unintended compared to those with equal number of sons and daughters (AOR 0.5, 95% CI 0.3–0.8) (Table 2).

**Table 2:** Multivariate logistic regression of sex composition and unintended pregnancy

Characteristic	OR	95% CI	*P*-Value
Sex Composition	1.1*	[1.0,1.2]	0.013
Equal number of sons and daughters (REF)			
Only daughters	0.7	[0.5,1.1]	0.098
Only sons	0.5**	[0.4,0.8]	0.001
More daughters than sons	1.1	[0.6,2.0]	0.756
More sons than daughters	1.6	[0.9,2.8]	0.128
Age	0.8**	[0.7,0.9]	0.000
15–19 (REF)			
20–24	0.5	[0.2,1.3]	0.177
25–29	0.5	[0.2,1.3]	0.156
30–34	0.2**	[0.1,0.6]	0.003
35–39	0.3*	[0.1,0.7]	0.010
40+	0.3*	[0.1,0.9]	0.027
Educational level	1.0	[0.8,1.1]	0.625
No education (REF)			
Primary	1.0	[0.7,1.5]	0.885
Middle/JSS	1.2	[0.8,1.7]	0.442
SSS/SHS/Vocational	0.9	[0.5,1.7]	0.678
Tertiary	1.1	[0.3,3.5]	0.909
Religion	1.0	[0.7,1.4]	0.933
Christian (REF)			
Muslim	0.7	[0.4,1.4]	0.368
Others	1.3	[0.6,3.2]	0.519
Parity	2.2**	[1.6,3.0]	0.000
1–2 (REF)			
3–4	1.4	[0.9,2.3]	0.120
5+	3.8**	[1.7,8.6]	0.001
Residence	1.1	[1.0,1.2]	0.306
Saltpond (REF)			
Biriwa	1.4	[0.8,2.3]	0.243
Anomabo	1.8*	[1.1,2.8]	0.017
Mankessim	1.2	[0.8,1.8]	0.377
Others	1.3	[0.9,1.9]	0.244
Occupation	0.9*	[0.8,1.0]	0.036
Fishmongers (REF)			
Farmers	1.2	[0.6,2.7]	0.608
Petty traders	0.9	[0.6,1.4]	0.767
Civil/public servants	0.5	[0.2,1.2]	0.128
Others	0.7	[0.4,1.2]	0.160
Marital Status	1.1	[1.0,1.3]	0.128
Ordinace (REF)			
Traditional	1.0	[0.7,1.7]	0.847
Engaged	0.7	[0.4,1.2]	0.217
Cohabiting	1.1	[0.6,2.0]	0.729
Single	2.2	[0.8,5.9]	0.118

*REF* Reference Category; * *p* < .05, ** *p* < .01; 95% confidence intervals in brackets

Women with five or more living children were more likely than those with 1 to 2 living children to classify their current pregnancy as unintended (AOR 3.8, 95% CI1.7–8.6). Likewise, women living in rural settings like Anomabo (AOR 1.8, 95% CI 1.1–2.8) and younger women (< 20 years) respectively, were more likely to report unintended pregnancies compared to those living in semi-urban settings (Saltpond) and older women (> 30 years) (Table 2). No significant association existed between respondents sex composition and intention to use postpartum family planning, however, whilst women resident in Anomabo were more likely to have intention to use postpartum family planning (AOR 1.8, 95% CI1.0–3.3), those from Mankessim were less likely to have intention to use postpartum family planning (AOR 0.6, 95% CI 0.4–0.9), compared to those from Saltpond respectively (Table 3).

**Table 3:** Multivariate logistic regression of sex composition and intention to use PPFP

Characteristic	OR	95% CI	*P*-value
Sex Composition	1.0	[1.0,1.1]	0.651
Equal number of sons and daughters (REF)			
Only daughters	1.1	[0.7,1.8]	0.582
Only sons	1.0	[0.6,1.6]	0.979
More daughters than sons	1.3	[0.7,2.5]	0.411
More sons than daughters	1.0	[0.5,1.8]	0.909
Age	1.0	[0.8,1.1]	0.555
15–19 (REF)			
20–24	1.7	[0.8,4.0]	0.200
25–29	1.9	[0.8,4.7]	0.118
30–34	1.6	[0.7,3.9]	0.308
35–39	1.3	[0.5,3.3]	0.614
45+	1.6	[0.5,5.3]	0.473
Educational level	0.9	[0.8,1.1]	0.271
No education (REF)			
Primary	1.6	[1.0,2.5]	0.051
Middle/JSS	1.4	[0.9,2.0]	0.153
SSS/SHS/Vocational	0.8	[0.4,1.5]	0.423
Tertiary	0.6	[0.2,2.1]	0.460
Religion	0.9	[0.6,1.2]	0.505
Christian (REF)			
Muslim	0.8	[0.4,1.6]	0.538
Others	1.0	[0.4,2.8]	0.951
Parity	1.2	[0.9,1.6]	0.308
1–2 (REF)			
3–4	1.1	[0.6,1.8]	0.799
5+	1.3	[0.6,3.1]	0.482
Residence	0.9	[0.8,1.0]	0.109
Saltpond (REF)			
Biriwa	0.7	[0.4,1.3]	0.255
Anomabo	1.8*	[1.0,3.3]	0.035
Mankessim	0.6*	[0.4,0.9]	0.015
Others	0.8	[0.5,1.2]	0.297
Occupation	0.9	[0.8,1.0]	0.072
Fishmongers (REF)			
Farmers	1.1	[0.5,2.5]	0.742
Petty traders	1.1	[0.7,1.7]	0.829
Civil/public servants	0.7	[0.3,2.0]	0.527
Others	0.7	[0.4,1.2]	0.217
Marital Status	1.1	[0.9,1.3]	0.314
Ordinace (REF)			
Traditional	1.4	[0.9,2.4]	0.145
Engaged	0.9	[0.5,1.7]	0.804
Cohabiting	1.4	[0.7,2.6]	0.323
Single	1.0	[0.4,2.7]	0.999

*REF* Reference Category; * *p* < .05, ** *p* < .01; 95% confidence intervals in brackets

## Discussion

Strong gender preference often stems from the requirements of lineage. Matrilineal and matrilocal societies may prefer couples to have more daughters, whilst strong patrilineal and patrilocal societies may prefer couples have more boys [16]. There is however a large body of evidence indicating that both sons and daughters are desired [3].The reported sex composition of living children was skewed towards males in this study. While 78% of the women had at least a son, 71% had at least a daughter with those having only sons exceeding those with only daughters by 6.3%. Also, a high proportion of women with more sons than daughters reported that their current pregnancies were unintended. These observations coupled with a sex ratio of 109:100 may infer that although both sexes are desired, some underlying son preference persisted.

The communities that the women are resident in (Anomabo, Biriwa, and Saltpond) are predominantly coastal in nature, with fishing as the main livelihood. Because the fishing industry is male dominated, with a high demand for males, sons may be preferred to ensure continuity of the business. Further investigations may be required to consolidate this fact. The inferred underlying son preference in this typical matrilineal system is consistent with findings in previous studies conducted in sub-Saharan Africa and other countries where male preference had been widely reported [14, 17, 18] and contrasts studies in Malawi and tribal societies of Meghalaya, India where preference for daughters was the norm in predominantly matrilineal areas [19, 20].

The highest proportion of unintended pregnancy was found among women with more sons than daughters. This implies that majority of women in this category did not want the pregnancy at the time it occurred or did not want it at all. The reasons may be that, they had satisfied the number and composition of their children. These findings are consistent with other studies in South Asia where women with more sons were less likely to want more children, and therefore any pregnancy that occurred may be unintended [3, 5, 6]. Unintended pregnancies, especially in sub-Saharan Africa, invariably end up in unsafe abortions whose associated health consequences and burdens disproportionately affect women. In many countries in sub-Saharan Africa, women’s access to safe abortion and post-abortion care for complications is hampered by restrictive laws, socio-cultural barriers, and inadequate resources to provide safe abortion [21]. These need to be addressed in order to prevent women from dying from unintended pregnancies.

In this study, women with only sons were significantly less likely to be carrying unintended pregnancies at the time of the survey. This implied that these women intended the pregnancies they were carrying. A similar finding was made by Calhoun and colleagues where families with only sons were significantly more likely to want more children and therefore any additional pregnancy may be intended [3]. For the women with only sons, any additional pregnancy may be intended to satisfy the sex composition of their children and to ensure continuity of the matrilineal inheritance system. This may be consistent with studies that showed that both girls and boys are valued in a system which is matrilineal but has patriarchal elements [3, 5].

This study also revealed that sex composition of children is not significantly associated with intention to use postpartumn contraceptives. This is not surprising because globally, research on the association between family sex composition and fertility behaviours such as fertility desires, intentions to use family planning and actual family planning use is not conclusive [3]. Intention to use postpartum family planning in this study was however influenced by residence in rural settings likeAnomabo because they reported the most unintended pregnancies compared to those resident in semi-urban settings like Saltpond.

### Strengths and limitations of study

The cross-sectional design was appropriate for the study considering the time constraints imposed by the limited funding. The fact that the respondents were selected from health facilities may affect the generalizability of the findings. The threat of selection bias was highly mitigated by ensuring that the data collectors explained the study objectives and their implications very well to the respondents before asking for consent. Some of the data collectors abandoned the study because of inadequate remuneration. New data collectors had to be trained to continue data collection. This brought about some delays in data analysis and reporting.

## Conclusions

The purpose of the study was to explore the existence of sex preference in a matrilineal inheritance system by examining the sex composition of living children and how it is associated with reproductive or fertility outcomes such as pregnancy intendedness and intention to use postpartum family planning. The persistence of more sons than daughters, high sex ratio and the high prevalence of unintended pregnancy among women with more sons than daughters, inferred son preference in a matrilineal inheritance system. Among the women with only sons, any additional pregnancy was intended to satisfy the sex composition of their children in order to consolidate and ensure continuity of the matrilineal inheritance system. Furthermore, sex composition of children was significantly associated with pregnancy intendedness and not postpartum family planning intentions. Women with more sons than daughters may have the highest unmet need for family planning. Postpartum family planning programming will require deeper understanding of the traditional and family contexts in which programmes are launched in order to ensure effective targeting.
